# Jottings

**Published:** 1910-03-05

**Authors:** 


### Jottings.

The Workpeople's Special Committee in aid of the York County Hospital has raised a sum of nearly £1,060 during the past year.

The annual general meeting of the Governors of the British Lying-in Hospital will be held at 5 p.m. on Tuesday, March 8.

The annual general meeting of the Hôpital et Dispensaire Française will be held at the hospital, 172 Shaftesbury Avenue, W.C., on Thursday, March 10, at 6 o'clock.

The annual meeting of the New Hospital for Women will be held at the hospital, 144 Euston Road, N.W., on Thursday, March 10, at 4 p.m., Mrs. Fawcett, LL.D., in the chair.

At the annual meeting of the Newcastle Maternity Hospital it was stated that the committee has decided to buy a site and build a new hospital, the present buildings being quite inadequate for the work.

If the Local Government Board should decide to add puerperal fever to the list of infectious diseases for which the Asylums Board are required to provide, the latter will be prepared to arrange for the reception of certified cases of this disease into the hospitals under their control.

The Right Hon. the Earl Cawdor has consented to preside at the annual meeting of the governors, donors, and subscribers of the London Homœopathic Hospital, Great Ormond Street, W.C., to be held in the board-room of the institution on Friday, March 11, at 3.30 p.m.

The Mount Vernon Hospital for Consumption and Diseases of the Chest, Hampstead and Northwood, will hold the annual court of Governors at the offices, No. 7 Fitzroy Square, London, W., on Wednesday, March 16, 1910, at 4 p.m., Captain Jessel, M.P., being in the chair.

The sanction of the Local Government Board is being asked by the Lewisham Guardians for the erection of a corrugated iron building for cases arising in the Workhouse and Infirmary requiring isolation. The scheme, estimated to cost about £370, has been spoken well of by the L.G.B. Inspector.

The Leeds Workpeople's Hospital Fund, though showing a falling-off last year, has a fine record in the collections made at various local hotels and public-houses, the income from this source amounting to £1,041 in 1908 and £1,469 in 1907. There is a balance of £8,884, however, from other quarters for the local charities.

The annual meeting of the Great Northern Central Hospital, Holloway Road, N., will be held on Thursday, March 10, at 5 p.m. On the agenda appears the following: "To consider the recommendation of the Committee of Management that the Surgeon to the Throat and Ear Department shall be a Fellow of the Royal College of Surgeons of England."

The annual report of the Liverpool Hospital for Cancer and Skin Diseases discloses the fact that during the past year extensive improvements and alterations have been undertaken at this institution. New operating, *x*-ray, and electrical rooms have been built, and a new laboratory has been equipped. The institutional returns show an adverse balance of £171 odd.

At the annual meeting of the Manchester Northern Hospital for Women and Children, held last week, the Mayor of Salford spoke strongly in favour of admitting paying patients into the wards, and stated that he thought it would be a bad day when institutions like the hospitals would have to come under the scope of municipal corporations. A new operating theatre is urgently needed for this hospital.

